# Amphotericin B Ocular Films for Fungal Keratitis and a Novel 3D-Printed Microfluidic Ocular Lens Infection Model

**DOI:** 10.3390/jof10110762

**Published:** 2024-11-02

**Authors:** Chrysi Rapti, Francis C. Luciano, Brayan J. Anaya, Bianca I. Ramirez, Baris Ongoren, María Auxiliadora Dea-Ayuela, Aikaterini Lalatsa, Dolores R. Serrano

**Affiliations:** 1 Pharmaceutics and Food Technology Department, Faculty of Pharmacy, Universidad Complutense de Madrid, Plaza Ramón y Cajal s/n, 28040 Madrid, Spain; xrisarapti1@gmail.com (C.R.); fluciano@ucm.es (F.C.L.); branaya@ucm.es (B.J.A.); biancram@ucm.es (B.I.R.); bongoren@ucm.es (B.O.); 2 Department of Pharmacy, Universidad Cardenal Herrera-CEU, CEU Universities, 46115 Alfara del Patriarca, Spain; mdea@uchceu.es; 3 CRUK Formulation Unit, School of Pharmacy and Biomedical Sciences, University of Strathclyde, John Arbuthnot Building, Robertson Wing, 161 Cathedral St, Glasgow G4 0RE, UK; 4 Instituto Universitario de Farmacia Industrial, Faculty of Pharmacy, Universidad Complutense de Madrid, 28040 Madrid, Spain

**Keywords:** keratitis, ocular films, amphotericin b, amphotericin B dimer, aggregation state, microfluidics, 3D printing

## Abstract

Fungal keratitis (FK), a severe eye infection that leads to vision impairment and blindness, poses a high risk to contact lens users, and *Candida albicans* remains the most common underpinning fungal pathogen in temperate climates. Patients are initially treated empirically (econazole 1% drops hourly for 24–48 h), and if there is no response, amphotericin B (AmB) 0.15% eye drops (extemporaneously manufactured to be stable for a week) are the gold-standard treatment. Here, we aim to develop a sustained-release AmB ocular film to treat FK with an enhanced corneal retention time. As there is a paucity of reliable in vitro models to evaluate ocular drug release and antifungal efficacy under flow, we developed a 3D-printed microfluidic device based on four chambers stacked in parallel, in which lenses previously inoculated with a *C. albicans* suspension were placed. Under the flow of a physiological fluid over 24 h, the release from the AmB-loaded film that was placed dry onto the surface of the wetted contact lenses was quantified, and their antifungal activity was assessed. AmB sodium deoxycholate micelle (dimeric form) was mixed with sodium alginate and hyaluronic acid (3:1 *w*/*w*) and cast into films (0.48 or 2.4%), which showed sustained release over 24 h and resulted in a 1.23-fold reduction and a 5.7-fold reduction in CFU/mL of *C. albicans*, respectively. This study demonstrates that the sustained delivery of dimeric AmB can be used for the treatment of FK and provides a facile in vitro microfluidic model for the development and testing of ophthalmic antimicrobial therapies.

## 1. Introduction

Infectious keratitis (IK) represents the leading cause of corneal blindness globally, with an estimated incidence of 2.5–799 cases per 100,000 population/year [1]. Subject to geographical and temporal variations, bacteria and fungi remain the most common causative organisms in patients with risk factors (contact lens wear, trauma, ocular surface disease, poor personal or environmental hygiene, lack of education, access to healthcare). Fungal keratitis (FK) poses diagnostic challenges (due to low and slow culture yield), has the propensity for deeper infections affecting the posterior cornea, can be treated by limited broad-spectrum antifungals for ocular use, especially in developing countries, and requires treatment with unlicensed off-label drugs. Patients are initially treated empirically with econazole 1% eye drops (hourly for first 24–48 h), and if there is no response, amphotericin B (AmB) 0.15% eye drops (specially manufactured, shelf-life of a week) are the gold-standard treatment. A concentration of 0.15% is used due to toxicity [2]. For FK, voriconazole 1% drops or natamycin have been used, but consultant monitoring is required. Treatment is combined with cycloplegic drops (atropine 1% drops twice daily or cyclopentolate 1% drops twice daily), and steroids are initiated at the discretion of the ophthalmologist, usually 48 h after topical antifungal therapy (dexamethasone sodium phosphate 0.1% drops, one drop daily). Even if the treatment is intensive, pathogens may still advance to corneal perforation in less than 48 h. Intracameral AmB (10 µg in 0.1 mL) may be given in cases where treatment response is poor, particularly in the presence of a hypopyon in the culture of the washings after an anterior chamber washout. Where secondary endophthalmitis develops, intravitreal AmB (5–10 µg in 0.1 mL) may be given, preceded by a vitreous biopsy where possible. Surgical interventions, such as therapeutic penetrating keratoplasty, may be considered [3].

FK is most frequently associated with filamentous fungi (*Fusarium* and *Aspergillus* spp.) in tropical latitudes and yeast (*Candida* spp.) in temperate climates [2]. Voriconazole has the lowest minimum inhibitory concentration (MIC) for *Aspergillus* spp. isolated from keratitis, and voriconazole and AmB have the lowest MICs versus *Fusarium* spp. [2]. The effectiveness of the antifungal agent is greatly affected by the achieved concentrations locally. In practice, topical antifungals are administered in different concentrations (e.g., natamycin 5%, while AmB is only 0.15%), which, if considered, means that natamycin has lower relative MICs than AmB for both *Fusarium* species and *Aspergillus* species. However, neither AmB nor natamycin penetrate well through an intact epithelium compared to topically applied voriconazole, which effectively penetrates even an intact corneal epithelium. Thus, delivery strategies are required to enhance the efficacy of broad-spectrum agents in treating FK.

AmB remains the gold-standard treatment for *C. albicans* spp. Infection begins when *C. albicans* attaches to epithelial surfaces, or implanted medical devices such as lenses, forming biofilms [4], and Candida biofilms display resistance to antifungals. Biofilm formation plays a crucial role, as it enhances the fungus’s ability to thrive in diverse environments while providing a protective barrier against antifungals and the body’s immune system [5]. AmB interacts with the ergosterol-forming pores in the fungal membrane, leading to apoptosis [6,7,8]. AmB, however, can also interact with mammalian cholesterol, resulting in toxicity; thus, dose adjustment is crucial. No ocular formulation is commercially available, which limits clinical use in FK. Specially manufactured AmB drops, prepared in hospital pharmacies, can only solubilise low AmB amounts (0.15%) and are only stable for 1 week, which increases the cost of therapy. Additionally, AmB suffers from low residence time, which limits its use against ocular biofilms [9] and permeation in high amounts locally. AmB’s aggregation state dictates its efficacy and safety [10]. The monomeric form of AmB has elevated toxicity and can be administered topically with cyclodextrins [11], while larger aggregates of AmB bind more selectively to ergosterol, thereby enhancing its selectivity and efficacy. Dimeric AmB (Fungizone with sodium deoxycholate AmB micelles), although effective, is not used for subconjunctival administration [2] due to toxicity attributable to deoxycholate [12]. AmB eyedrops have very limited residence and, hence, sustained AmB ocular release would be ideal for FK treatment.

Microfluidics is a dynamic field focused on the precise manipulation of miniature fluid volumes and is emerging as a promising new technology in ophthalmology. The unique anatomical constraints of the eye, with its intricate structures and small size, in addition to the contained, precious, yet low volume of ocular fluids in the eye (i.e., aqueous humor, tear film, vitreous humor, cerebrospinal fluids, and blood), present challenges for traditional approaches to drug development and delivery and disease diagnosis. In this respect, microfluidics and lab-on-a-chip technologies hold the potential to revolutionize treatment strategies and improve outcomes for patients with a range of eye conditions, including infections, glaucoma, cataract, dry eye, diabetic retinopathy, age-related macular degeneration, and myopia [13,14,15,16].

**Figure 1:**
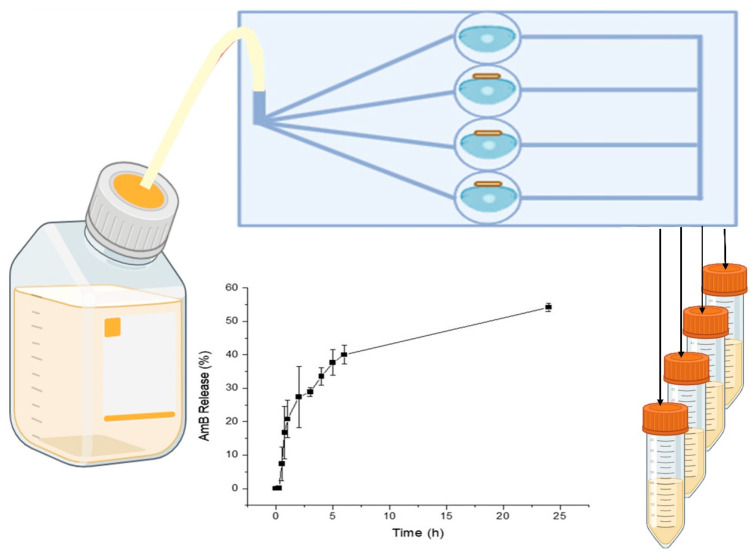
Schematic representation of 3D-printed microfluidic chip to assess release from film under continuous flow.

In this work, a sustained-release AmB formulation is able to increase the ocular residence time to effectively eradicate ocular fungal infections. AmB was formulated as a biocompatible ocular film able to control drug release over 24 h and to eliminate *C. albicans* from contact lenses. To test our hypothesis, we developed a novel 3D-printed microfluidic chip (Figure 1) that allowed continuous physiological fluid circulation via individual stacked chambers where contact lenses inoculated with *C. albicans* were placed. AmB-loaded ocular films were placed dry on the wetted surface of the lenses and the release after 24 h and antifungal efficacy were measured.

## 2. Materials and Methods

### 2.1. Materials

Amphotericin B (≥95% HPLC) was purchased from Azelis (Barcelona, Spain). Sodium alginate (Ph. Eur. Grade, >200 kDa) was purchased from Carlo Erba (Barcelona, Spain). Hyaluronic acid (417.3 Da) was acquired from Sigma-Aldrich^®^ (Madrid, Spain). Contact lenses (Dailies^®^, AquaComfort Plus, Nelficon AII, non-ionic, polyvinyl alcohol: hydroxypropylmethylcellulose: polyethylene glycol: n-formylmethyl acrylamide (PVA:HPMC:PEG:FMA), 69% water content, oxygen permeability: 26 Dk/t) were purchased from Alcon (Madrid, Spain). Phosphoric acid (85%) (H_3_PO_4_), sodium hydroxide (NaOH), and solvents were of analytical and HPLC grade. They were sourced from Panreac S.A. (Barcelona, Spain) and used without purification. Anycubic LCD UV 405 nm rapid photopolymer 3D Resin and the Anycubic Photon Mono X (LCD-based SLA printer, 405 nm light source, 0.05 mm 3840 × 2400 XY resolution, 0.01 mm Z resolution, 192 × 120 × 245 mm build volume) were purchased from Anycubic^®^ (Shenzhen, China).

### 2.2. Preparation of AmB-Loaded Ocular Films

Lyophilized AmB dimer was prepared as previously described [17]. Briefly, 50 mg of AmB was dispersed in 5 mL of deionized water. Sodium deoxycholate (41 mg), dibasic sodium phosphate (10 mg), and monobasic sodium phosphate (0.9 mg) were added. The pH was adjusted to pH 12.0 using NaOH 2 M. Once the AmB was dissolved, the pH was reduced to 7.4 by adding H_3_PO_4_ (diluted up to 10% *v*/*v*) and deionized water was added to achieve a concentration of 5 mg/mL. The dimeric AmB formulation was frozen (−40 °C) and lyophilized (Telstar, Barcelona, Spain).

AmB lyophilized dimer (5 mg or 25 mg) was added to 750 mg of sodium alginate and 250 mg of hyaluronic acid in 30 mL of deionized water. Sodium alginate, a biocompatible water-soluble salt of alginic acid [18], and hyaluronic acid (HA), able to maintain adhesion even at low polymer concentrations [19], were chosen as film formers. The solution was cast into five Petri dishes and left to dry protected from light at room temperature for 72 h until a thin film was obtained. The theoretical drug loading was 4.8 and 24 µg of AmB/mg of film, respectively.

### 2.3. Physicochemical Characterization of AmB-Loaded Ocular Films

The AmB film (1.5 mg, n = 3) was weighed, dispersed in 1 mL of AmB HPLC mobile phase, and vortexed over 5 min prior to the high-performance liquid chromatography (HPLC) quantification of AmB [8]. Analysis was undertaken using a Varian Prostar 230 Solvent Delivery Module, a Varian Prostar 410 Autosampler, and a Varian Prostar 310 UV-visible Detector (Varian^®^, Palo Alto, CA, USA) attached to a Thermo Scientific BDS Hypersil C18 (200 mm × 4.6 mm, 5 µm) column at room temperature. The mobile phase consisted of acetonitrile, glacial acetic acid, and deionized water in a 52:4.3:43.7 ratio (*v*/*v*), and the flow rate and injection volume were set at 1 mL/min and 50 µL, respectively. Detection was set at 406 nm, and data collection and processing were performed using the Galaxie Chromatography Data System (Varian^®^, CA, USA).

The particle size, polydispersity, and zeta potential of the dispersion in aqueous media (0.1 mg/mL) were determined using a Zetasizer (Malvern Instruments, Malvern, UK), as previously described [20]. Polystyrene standards (100 nm) were evaluated to ensure the accuracy of the measurements.

Fourier-transformed infrared spectra were recorded using a Nicolet Nexus 670–870 (Thermofisher^®^, Madrid, Spain) in the range of 400–4000 cm^−1^ with a 1 nm step scan. Data were analysed using Spectragryph software (version 1.2.9, Oberstdorf, Germany).

Powder X-ray diffraction (PXRD) analysis was conducted using a Philips^®^X’Pert-MPD X-ray diffractometer (Malvern Panalytical^®^; Almelo, The Netherlands) equipped with Ni-filtered Cu Kα radiation (1.54 Å) (Tokyo, Japan), operated at a tube voltage of 30 kV and a tube current of 25 mA. PXRD patterns were captured at a step scan rate of 0.05°/s, ranging from 5° to 40° on the 2-theta scale (n = 3) [21].

Differential scanning calorimetry (DSC) and thermogravimetric analysis (TGA) were performed simultaneously on an SDT Q600 instrument (TA Instruments, Elstree, UK) with nitrogen as the purge gas. Samples (4–6 mg) were weighted and heated (10 °C/min) to between 25 °C and 500 °C. The instrument was calibrated with indium as the reference standard.

### 2.4. Adhesion and Burst Strengths of AmB-Loaded Ocular Films

The in vitro adhesive and burst strengths of the AmB-loaded films were evaluated using the Texture Analyzer TA.XT Plus C (Stable Micro Systems Ltd., Surrey, UK), utilizing a cylindrical probe with a diameter of 0.5 inches (p/0.5) and a film support rig (HDP/FDR). To measure adhesive strength, the force required to detach the film from the commercial contact lenses was determined. The contact lens was securely mounted onto the base of the texture analyser. The film was attached to a cylindrical probe with a diameter of 0.5 inches (p/0.5). The probe was then driven into the contact lens at a constant speed of 0.5 mm/s. Once the probe was in contact with the contact lens, a 49 mN force was applied for 5 s, and then the probe was detached at a post-test speed of 10 mm/s. Data were collected at a rate of 200 points per second (PPS). The area under the curve (AUC) of the force–distance plot was used to quantify the adhesion of the film to the contact lenses using the Exponent software (version 8.0.14.0). The results were plotted using Origin 2021 (OriginLab Corporation, Northampton, MA, USA).

For the burst strength, the film was attached to the film support rig (HDP/FDR, Stable Microsystems). The force required to break the film was measured using a 5 mm spherical stainless-steel ball probe with a probe adapter that was connected to the load cell. A film (35 × 15 mm) was placed in the film support rig and the moving probe reached the surface of the film with a pre-test speed of 0.5 mm/s, a test speed of 1 mm/s, and a post-test speed of 10 mm/s. The penetration depth was 10 mm. The force applied had a trigger load of 5 N and the maximum force (mN), travel distance (mm), and area under the curve (mN mm) were recorded at a rate of 200 points per second (PPS). Data were analysed using Exponent software (version 8.0.14.0).

### 2.5. Particle Morphology upon Film Disintegration in Aqueous Media

After film (5 mg) reconstitution in deionized water (5 mL), a drop was deposited onto a Formvar/carbon-coated grid, and any excess sample was removed using Whatman No. 1 filter paper. The release of AmB micelles and particle morphology were evaluated after negative staining with 1% *w*/*v* phosphotungstic acid solution using a transmission electron microscope (TEM) (JEM 1400 plus, JEOL, Tokyo, Japan, with an acceleration voltage of 120 kV). Lyophilized AmB dimer was also reconstituted in deionized water (5 mg/mL) and analysed in the same manner for comparison.

### 2.6. Design and 3D Printing of In Vitro Microfluidic Devices

Anycubic LCD UV 405 nm rapid photopolymer 3D resin (translucent green) was chosen to allow for a balance between optical transparency and reduced light bleeding. The microfluidic chip was designed using Tinkercad (Autodesk^®^, Mill Valley, CA, USA). The chip was designed with a rectangular shape (length: 70 mm, width: 65, height: 32 mm), featuring four circular open chambers (opening diameter: 15 mm, height: 13 mm) for contact lens placement. These chambers were connected within the chip via an upward-facing inlet port channel with a 2 mm diameter for the media to circulate inside the chip to supply all the chambers and to ensure no blockages by microorganisms after inoculation. For media exit, each chamber was connected to its own channel that exited the chip through a downward-facing port, also with a 2 mm diameter. The four downward-facing outlet ports were spaced apart, enabling the easy and separate collection of media from each chamber. The final microfluidic chip design was exported into a standard tessellation language (.stl) digital file.

The first eight layers underwent extended UV light exposure (60 s, Anycubic^®^ Photon Mono X SLA printer) to ensure strong adhesion to the platform, while subsequent layers received shorter exposure (3 s), followed by a 1 s interval where the UV light was deactivated to avoid unintended curing [22]. Each subsequent layer had a 0.05 mm thickness. Washing and curing were performed (Anycubic^®^ Wash & Cure Machine 2.0) by immersing the chip in isopropyl alcohol 70% for 15 min and placing it into the curing mode for 60 min. The geometry was visualized with an iPhone 10 with a 12-megapixel camera (f/1.8, 1.22-micron) (Apple, Cupertino, CA, USA).

### 2.7. Film Release and AmB Antimicrobial Efficiency

AmB release from the ocular films was evaluated simultaneously along with its antifungal efficacy. Yeast isolates (*C. albicans* CECT 1394) were cultured on Sabouraud dextrose agar at 30 °C for 48 h to ensure viability and the absence of contamination. Yeast suspensions (5 mL in 0.9% NaCL, 0.1 absorbance at 600 nm) were used to inoculate each of the four contact lenses (Dailies^®^, AquaComfort Plus, Hongkong, China) over 1 h. Lenses were blotted using Whatman N°1 filter paper and transferred into one of the four microfluidic chambers. AmB-loaded films (1.5 mg) were adhered on the surface of three lenses, while one lens was used as the infected untreated control, and the chips were incubated (S-Bt Smart Biotherm incubator Biosan^®^, Proquinorte, Madrid, Spain). The inlet port was connected via a silicone tube (inner Ø 2 mm) to a media glass bottle containing yeast extract peptone dextrose (YPD) sterile broth, and the media were circulated using a peristaltic pump (Cole Palmer Masterflex L/S 7518-00, Vernon Hills, IL, USA) at 350 µL/min over 24 h. Effluxed media were collected at different points (15, 30, 45, 60, 120, 180, 240, 300, 360, and 1440 min), and 100 µL from each time point was mixed with 900 µL in the HPLC mobile phase, prior to vortexing and centrifugation (5000 rpm, 10 min), for AmB quantification by HPLC, as above. Experiments were repeated in triplicate for both AmB-loaded films (0.48 and 2.4%).

After 24 h, the lenses were removed, placed in 5 mL of 0.9% NaCl solution, and sonicated (Ultrasons-H, J.P. Selecta^®^, 200 watt, 10 min). Samples were diluted in different ratios of 1:4, 1:8, 1:12, 1:16, and 1:32 *v*/*v*, from which 0.1 mL was spread on a Petri dish with Mueller Hinton media using a sterile bent glass rod and incubated at 30 °C for 48 h (S-Bt Smart Biotherm incubator), prior to counting the colonies grown and calculating the colony-forming units.

### 2.8. In Vitro Cytotoxicity in APRE (Arising Retinal Pigment Epithelia) Cells

ARPE-19 (CRL-2302) is a spontaneously arising retinal pigment epithelia (RPE) cell line derived from the normal eyes of a 19-year-old male who died from head trauma in a motor vehicle accident. ARPE-19 cells were purchased from ATCC and were cultured in F12 medium supplemented with 15 mM HEPES, 1.6 mM L-glutamine, 1.2 g/L NaHCO_3_, 3 g/L glucose, 10% heat-inactivated Foetal Bovine Serum (FBS), 1000 U/L of penicillin, and 100 mg/L of streptomycin in 25 mL culture flasks at 37 °C, 5% CO_2_.

The assay was carried out as previously described [19,20,21,22,23], with certain modifications. Briefly, cells were seeded (4 × 10^4^ cells/well) in 96-well flat-bottom microplates with 100 μL of F12 medium. The plates were then incubated at 37 °C with 5% CO_2_ for 48 h. When the cultures were approximately 75% to 80% confluent, the medium was replaced by different concentrations of AmB or AmB film in 200 μL of medium and exposed for another 48 h. Growth controls and the signal-to-noise measurements were also included. Afterwards, a volume of 20 μL of the 2.5 mM resazurin solution was added, and the plates were returned to the incubator for another 3 h to evaluate cell viability. The reduction in resazurin was determined by fluorometry. Each concentration was assayed three times. The cytotoxicity effect of the compounds was defined as a 50% reduction in the cell viability of the treated culture cells with respect to the untreated culture (CC_50_), and was calculated using a multinomial probit analysis incorporated in SPSS software v21.0.

### 2.9. Statistical Analysis

Two-sample *t*-tests using Minitab 19 (Minitab Ltd., Coventry, UK) were undertaken and significance was set at *p* < 0.05.

## 3. Results

### 3.1. Physicochemical Characterization of AmB-Loaded Ocular Films

The AmB dimer had a concentration 4.975 ± 0.015 mg/mL, and films based on low or high AmB loading had concentrations of 0.476 ± 0.004% and 2.357 ± 0.012% *w*/*w*, respectively. The average size of the AmB dimer in the solutions was 70 ± 5 nm, while for the AmB-loaded film, after reconstitution in aqueous media, this was 66 ± 7 nm and 68 ± 3 nm for 0.48 and 2.4% loadings, respectively. No statistically significant differences were observed between the dimer and the films, which suggests that the incorporation of AmB into the film did not alter the particle size of the micellar suspension.

**Figure 2:**
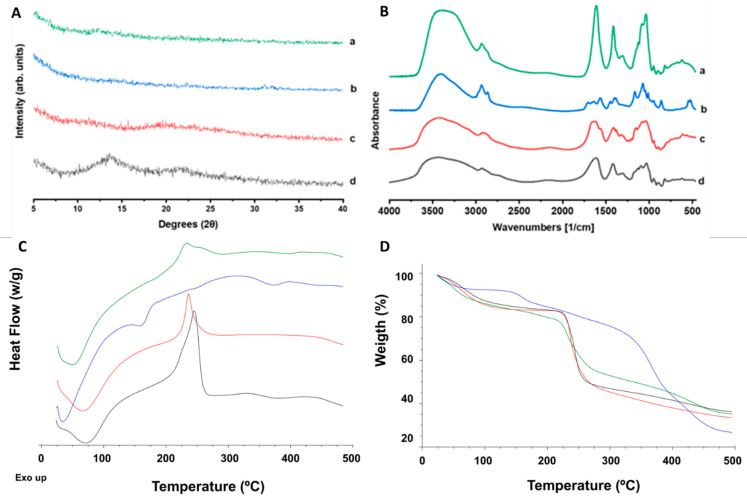
Solid-state characterization analysis (top) (**A**): X-ray diffraction pattern: (a) AmB-loaded film, (b) lyophilized AmB dimer, (c) unprocessed hyaluronic acid, and (d) unprocessed sodium alginate. (**B**) Fourier-transform infrared (FTIR) spectra: (a) AmB-loaded film, (b) lyophilized AmB dimer, (c) unprocessed hyaluronic acid, and (d) unprocessed sodium alginate. Thermal characterization analysis (bottom): (**C**) differential scanning calorimetry (DSC) analysis and (**D**) thermogravimetric analysis (TGA) of AmB film (green line), AmB dimer (blue line), hyaluronic acid (red line), and sodium alginate (black line).

The XRD pattern of both the lyophilized AmB dimer and the AmB-loaded film displayed a characteristic amorphous halo (Figure 2A), while AmB showed a crystalline nature [24]. Drug amorphization enhances solubility in aqueous media, which is important for ocular applications.

The FTIR spectra show broad bands for the films, which correlates with their amorphous nature. The characteristic bands for the AmB-loaded films at 1611 cm^−1^ and 1411 cm^−1^ are attributed to C=O stretching and O-H bending, respectively, due to the presence of both sodium alginate and hyaluronic acid polymeric chains and the higher presence of carboxylic and alcohol groups. The AmB dimer also exhibits a characteristic carboxylic group (C=O band at 1708 cm^−1^) and a primary amine (band at 1546 cm^−1^) responsible for the interaction with sodium deoxycholate. The bands are shifted compared to AmB (unprocessed), indicating hydrogen bonding with sodium deoxycholate [8]. The strong AmB dimer bands at 2858 cm^−1^ and 2938 cm^−1^ are attributed to O-H stretching (intramolecular bonds) and N-H stretching, and were also identified in the AmB-loaded film with a lower intensity.

The DSC analysis showed a dehydration broad peak up to 100 °C for all the components (Figure 2C). Sodium alginate and hyaluronic acid thermographs showed no endothermic events corresponding to melting events, but a marked exothermic peak after 200 °C was observed, attributed to degradation and responsible for significant weight loss (Figure 2D). In the case of hyaluronic acid, degradation occurs before melting, which has been reported at 241 °C [25]. The AmB dimer showed two endothermic events starting at 150 and 350 °C attributed to the melting of AmB and sodium deoxycholate, respectively [26]. Both peaks are attributed to weight loss and degradation within the same temperature range. The AmB-loaded film did not exhibit any endothermic event, but only a mild exothermic peak corresponding to the partial degradation of the hyaluronic acid and sodium alginate. The TGA analysis of the AmB films indicated a similar profile to the film-forming polymers and lower thermal stability than the lyophilized AmB dimer (Figure 2D).

**Figure 3:**
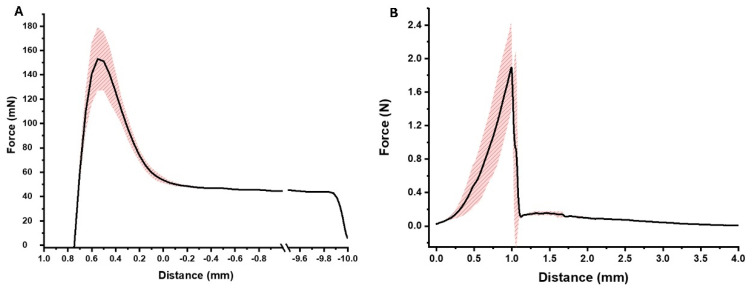
Mechanical properties of AmB-loaded films. (**A**) Adhesion to contact lens; (**B**) burst strength of AmB-loaded films. Shadow area in red indicates the standard deviation between measurements (n = 3).

The adhesive strength of the film to the ocular lenses, and ideally the surface of the cornea, and the burst strength are illustrated in Figure 3. AmB films adhered to the lenses and required a force of 153 ± 26 mN for detachment (Figure 3A). The film showed adequate burst strength (1.89 ± 0.53 N), but less flexibility [short travel distance to the maximum force (0.99 ± 0.04 mm)] (Figure 3B).

**Figure 4:**
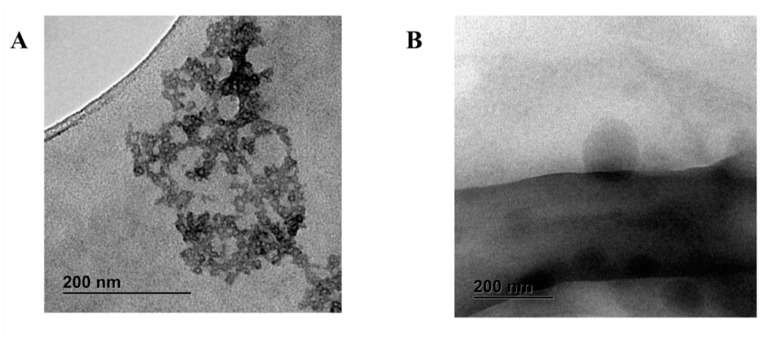
Transmission electron microscopy (TEM) microphotographs. (**A**) AmB dimer; (**B**) AmB film stained with 1% phosphotungstic acid. Bar: 200 nm.

The AmB dimer was present in micelles below 100 nm in size, aggregating in larger micellar clusters (Figure 4A). After reconstituting the AmB films in water, spherical particles also below 100 nm in size were present, indicating the stability of AmB dimeric micelles upon film rehydration and release (Figure 4B).

### 3.2. Design and 3D Printing of Microfluidic Devices

**Figure 5:**
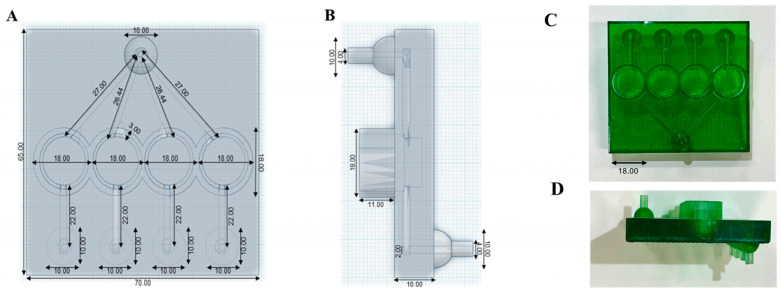
Design and dimensions of the 3D-printed microfluidic device. (**A**) Top view of the 3D-printed chip design. (**B**) Side view of the 3D-printed chip design (mm). (**C**) Top view of the 3D-printed ocular chip. (**D**) Side view of the 3D-printed ocular chip (mm). Dimensions are expressed in mm.

The 3D-printed ocular chip design is summarized in Figure 5, and the dimensions of the 3D-printed chip reliably matched the initial design based on a biocompatibility assessment after adding the curing resin.

### 3.3. Evaluation of AmB-Loaded Film Release and Antimicrobial Efficacy

**Figure 6:**
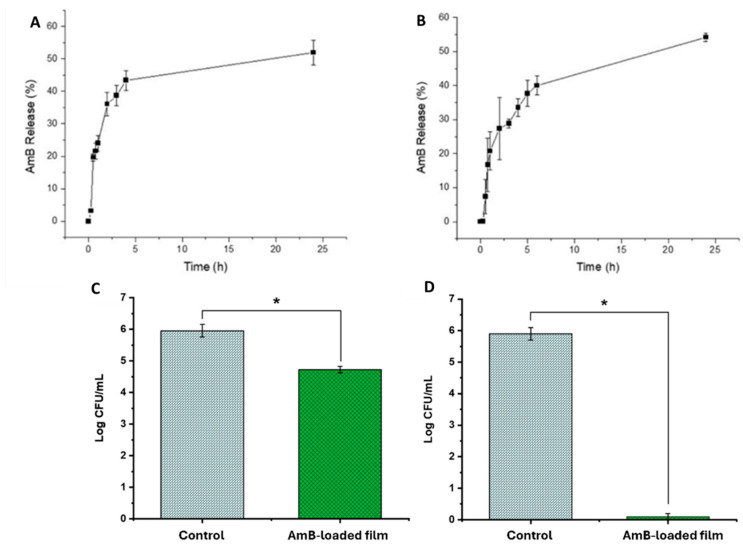
AmB release from (**A**) AmB film (0.48% *w*/*w*) and from (**B**) AmB film (2.4% *w*/*w*), and in vitro antifungal activity of the (**C**) AmB film (0.48% *w*/*w*) and (**D**) AmB film (2.4% *w*/*w*) versus control (blank untreated contact lenses). Statistical significant differences (p< 0.05) are depicted with *.

AmB release from the films (0.48 and 2.4% drug loading) was gradual and sustained over a 24 h period, with faster release kinetics over the first 4 h eliciting therapeutic AmB levels (>15 µg/mL, Figure 6A,B). A controlled release profile is required for the effective treatment of FK, minimizing the need for frequent administration while maintaining therapeutic levels over an extended period. No difference was observed between the 0.48 and 2.4% drug-loaded films, indicating that AmB dose in the films can be tailored to patients’ needs.

The in vitro antifungal efficacy was evaluated using the 3D-printed microfluidic device. Statistically significant differences (*p* < 0.05) were observed between the AmB-treated lenses versus the non-treated ones (0.48% in Figure 6C and 2.4% in Figure 6D). When the drug loading was low (0.48%), the log CFU/mL was reduced by 1.23-fold, while the reduction in microbial activity was 5.7-fold (5.7 log CFU/mL to 0 log CFU/mL) when the AmB loading was increased 5-fold in the film (2.4%).

### 3.4. Evaluation of AmB-Loaded Films’ In Vitro Cytotoxicity

AmB ocular films (2.4% *w*/*w*) were assessed in the context of APRE-19 cells and demonstrated a three-fold higher cytotoxic concentration compared to AmB alone (Table 1). The higher safety and tolerability of the AmB film compared to AmB solutions are in accordance with previously published microneedle AmB patches for intracorneal fungal infections [27].

## 4. Discussion

Here, we have demonstrated a novel AmB film for FK and the first microfluidic device to allow for the simultaneous in vitro evaluation of antimicrobial release and activity. Only a few organ-on-a-chip examples are available to evaluate drug permeability, distribution in ocular tissues, and drug release. Models like the “blinking eye-on-a-chip”, aiming to replicate tear film dynamics and the mechanical shear forces exerted on the corneal epithelium during blinking, are under development to understand the interactions between the ocular surface and various medical devices [28]. A lens-on-a-chip system has been used to assess the disinfection efficacy of lens care solutions, as well as the differences between observed biofilms and planktonic bacteria [29]. The proposed microfluidic device can be easily adapted to allow the integration of corneal cells for cytotoxicity and permeability measurements with existing organ-on-a-chip technologies. Ex vivo models of fungal keratitis [30] rely on excised rabbit or human tissue inoculated with microorganisms for assessment. Ethical permission is required to work with rabbit models or human cornea; the latter is extremely rare and in short supply for transplants in patients with damaged corneas. Consequently, synthetic or animal tissues are utilized routinely in patients to overcome this short supply. Microfluidic models, such as the one proposed, can overcome these limitations and enable a faster translation of novel agents and advanced delivery systems from the lab to patients.

A dimeric AmB state was selected based on its lower toxicity against mammalian cells and its higher antifungal efficacy [17], and was embedded in a film formed by negatively charged biocompatible polymers (sodium alginate, hyaluronic acid) known to increase corneal adhesion [31,32,33] to the positively charged corneal epithelium [34]. Sodium alginate consists of β-D-mannuronate and α-L-guluronate residues with a carboxyl group in the C-6 position that is partially ionized at neutral pH, giving alginate films their characteristic negative charge [35]. As the adhered film dissolves and AmB is gradually released, the drug’s residence time is increased and the frequency of administration and occurrence of resistance are reduced, improving patient compliance and outcomes. An AmB microneedle ocular patch has recently been tested in an FK model [27], but its complexity of fabrication, poor physicochemical stability, and limited mechanical strength make its clinical translation challenging. Our AmB-loaded films (2.4% *w*/*w*) demonstrated comparable, if not higher biocompatibility than these previously published AmB patches [27]. They were also able to demonstrate sustained AmB release over 24 h and show strong antifungal activity against Candida albicans. FK in contact lens wearers is associated with cornea microabrasions, which can elicit intravitreal drug levels, and retinal cells can be affected by fungi, leading to complications such as endophthalmitis. Typically, AmB in higher doses than 5–10 µg (although reports above 1 µg are available) is linked to significant retinal damage, which can be ameliorated by the use of sustained-release AmB-loaded films [36,37,38]. This first-of-its-kind in vitro microfluidic device is able to not only assess drug release and antifungal efficacy potential using clinical isolates, but can—with minor modifications—also assess drug permeability via the organ-on-a-chip technology described in the literature [22,39,40,41,42,43]. Thus, a limitation of our current model is that the inoculation of the infectious microorganism occurs on the surface of contact lenses, and further proof-of-concept data on cell monolayers or 3D models of corneas are required. Notably, 3D-printed chips offer ease of manufacture and facile customization, but compared to glass or polydimethylsiloxane chips, they are less optically clear and require biocompatible resins to be employed with minimal, if any, leaching [44].

## 5. Conclusions

In conclusion, this study provides proof-of-principle experiments towards an AmB film for FK and describes an in vitro versatile model to evaluate ocular antifungal dose, release, permeability, efficacy, and toxicity. This may revolutionize personalized medicine and improve patient outcomes in the field of ophthalmology. Further in vitro and in vivo studies will be beneficial in validating the technology towards clinical and laboratory testing prototypes.

## Figures and Tables

**Table 1:** In vitro cytotoxicity of AmB and AmB ocular films in APRE-19 cells.

Formulations	CC_50_ (µg/mL)
AmB	30.06 ± 0.98
AmB film (2.4% *w*/*w*)	101.13 ± 10.17

## Data Availability

The original contributions presented in the study are included in the article, further inquiries can be directed to the corresponding authors.

## References

[1] Ting D.S.J., Galal M., Kulkarni B., Elalfy M.S., Lake D., Hamada S., Said D.G., Dua H.S. (2021). Clinical Characteristics and Outcomes of Fungal Keratitis in the United Kingdom 2011–2020: A 10-Year Study. J. Fungi.

[2] Lalitha P., Shapiro B.L., Srinivasan M., Prajna N.V., Acharya N.R., Fothergill A.W., Ruiz J., Chidambaram J.D., Maxey K.J., Hong K.C. (2007). Antimicrobial susceptibility of Fusarium, Aspergillus, and other filamentous fungi isolated from keratitis. Arch. Ophthalmol..

[3] Hoffman J.J., Arunga S., Mohamed Ahmed A.H.A., Hu V.H., Burton M.J. (2022). Management of Filamentous Fungal Keratitis: A Pragmatic Approach. J. Fungi.

[4] Watchaputi K., Jayasekara L., Ratanakhanokchai K., Soontorngun N. (2023). Inhibition of cell cycle-dependent hyphal and biofilm formation by a novel cytochalasin 19,20-epoxycytochalasin Q in Candida albicans. Sci. Rep..

[5] Yuste I., Luciano F.C., Anaya B.J., Sanz-Ruiz P., Ribed-Sanchez A., Gonzalez-Burgos E., Serrano D.R. (2023). Engineering 3D-Printed Advanced Healthcare Materials for Periprosthetic Joint Infections. Antibiotics.

[6] Fernández-García R. (2022). Self-assembling, supramolecular chemistry and pharmacology of amphotericin B: Poly-aggregates, oligomers and monomers. J. Control. Release.

[7] Fernandez-Garcia R., de Pablo E., Ballesteros M.P., Serrano D. (2017). R Unmet clinical needs in the treatment of systemic fungal infections: The role of amphotericin B and drug targeting. Int. J. Pharm..

[8] Serrano D.R., Lalatsa A., Dea-Ayuela M.A., Bilbao-Ramos P.E., Garrett N.L., Moger J., Guarro J., Capilla J., Ballesteros M.P., Schatzlein A.G. (2015). Oral particle uptake and organ targeting drives the activity of amphotericin B nanoparticles. Mol. Pharm..

[9] Serrano D.R., Ruiz-Saldana H.K., Molero G., Ballesteros M.P., Torrado J.J. (2012). A novel formulation of solubilised amphotericin B designed for ophthalmic use. Int. J. Pharm..

[10] Serrano D.R., Hernandez L., Fleire L., Gonzalez-Alvarez I., Montoya A., Ballesteros M.P., Dea-Ayuela M.A., Miro G., Bolas-Fernandez F., Torrado J.J. (2013). Hemolytic and pharmacokinetic studies of liposomal and particulate amphotericin B formulations. Int. J. Pharm..

[11] Ruiz H.K., Serrano D.R., Dea-Ayuela M.A., Bilbao-Ramos P.E., Bolas-Fernandez F., Torrado J.J., Molero G. (2014). New amphotericin B-gamma cyclodextrin formulation for topical use with synergistic activity against diverse fungal species and *Leishmania* spp.. Int. J. Pharm..

[12] Cannon J.P., Fiscella R., Pattharachayakul S., Garey K.W., De Alba F., Piscitelli S., Edward D.P., Danziger L.H. (2003). Comparative toxicity and concentrations of intravitreal amphotericin B formulations in a rabbit model. Invest. Ophthalmol. Vis. Sci..

[13] Peng Z., Zhou L., Wai Wong J.K., Chan Y.K. (2020). Eye-on-a-chip (EOC) models and their role in the future of ophthalmic drug discovery. Expert Rev. Ophthalmol..

[14] Bai J., Fu H., Bazinet L., Birsner A.E., D’Amato R.J.A. (2020). A Method for Developing Novel 3D Cornea-on-a-Chip Using Primary Murine Corneal Epithelial and Endothelial Cells. Front. Pharmacol..

[15] Li Q., Wong H.L., Ip Y.L., Chu W.Y., Li M.S., Saha C., Shih K.C., Chan Y.K. (2023). Current microfluidic platforms for reverse engineering of cornea. Mater. Today Bio.

[16] Wright C.B., Becker S.M., Low L.A., Tagle D.A., Sieving P.A. (2020). Improved Ocular Tissue Models and Eye-On-A-Chip Technologies Will Facilitate Ophthalmic Drug Development. J. Ocul. Pharmacol. Ther..

[17] Espada R., Valdespina S., Alfonso C., Rivas G., Ballesteros M.P., Torrado J.J. (2008). Effect of aggregation state on the toxicity of different amphotericin B preparations. Int. J. Pharm..

[18] Ahmad S., Tanwee M.S. (2023). Antimicrobial gum based hydrogels as adsorbents for the removal of organic and inorganic pollutants. J. Water Process Eng..

[19] Snetkov P., Zakharova K., Morozkina S., Olekhnovich R., Uspenskaya M. (2020). Hyaluronic Acid: The Influence of Molecular Weight on Structural, Physical, Physico-Chemical, and Degradable Properties of Biopolymer. Polymers.

[20] Lalatsa A., Emeriewen K., Protopsalti V., Skelton G., Saleh G.M. (2016). Developing transcutaneous nanoenabled anaesthetics for eyelid surgery. Br. J. Ophthalmol..

[21] Celi S.S., Fernandez-Garcia R., Afonso-Urich A.I., Ballesteros M.P., Healy A.M., Serrano D.R. (2023). Co-Delivery of a High Dose of Amphotericin B and Itraconazole by Means of a Dry Powder Inhaler Formulation for the Treatment of Severe Fungal Pulmonary Infections. Pharmaceutics.

[22] Kara A., Vassiliadou A., Ongoren B., Keeble W., Hing R., Lalatsa A., Serrano D.R. (2021). Engineering 3D Printed Microfluidic Chips for the Fabrication of Nanomedicines. Pharmaceutics.

[23] Youn J., hoi J.H., Lee S., Lee W., Lee S.W., Kim W., Song Y., Tumursukh N.E., Song J.E., Khang G. (2022). Fabrication and Evaluation of Gellan Gum/Hyaluronic Acid Hydrogel for Retinal Tissue Engineering Biomaterial and the Influence of Substrate Stress Relaxation on Retinal Pigment Epithelial Cells. Molecules.

[24] Serrano D.R., Fernandez-Garcia R., Mele M., Healy A.M., Lalatsa A. (2019). Designing Fast-Dissolving Orodispersible Films of Amphotericin B for Oropharyngeal Candidiasis. Pharmaceutics.

[25] Lopez K.M., Ravula S., Pérez R.L., Ayala C.E., Losso J.N., Janes M.E., Warner I.M. (2020). Hyaluronic Acid-Cellulose Composites as Patches for Minimizing Bacterial Infections. ACS Omega.

[26] Rolon M., errano D.R., Lalatsa A., de Pablo E., Torrado J.J., Ballesteros M.P., Healy A.M., Vega C., Coronel C., Bolas-Fernandez F. (2017). Engineering Oral and Parenteral Amorphous Amphotericin B Formulations against Experimental Trypanosoma cruzi Infections. Mol. Pharm..

[27] Albadr A.A., Tekko I.A., Vora L.K., Ali A.A., Laverty G., Donnelly R.F., Thakur R.R.S. (2022). Rapidly dissolving microneedle patch of amphotericin B for intracorneal fungal infections. Drug Deliv. Transl. Res..

[28] Manafi N., Shokri F., Achberger K., Hirayama M., Mohammadi M.H., Noorizadeh F., Hong J., Liebau S., Tsuji T., Quinn P.M.J. (2021). Organoids and organ chips in ophthalmology. Ocul. Surf..

[29] Kravchenko S.V., Myasnikova V.V., Sakhnov S.N. (2023). Application of the organ-on-a-chip technology in experimental ophthalmology. Vestn. Oftalmol..

[30] Pinnock A., Myasnikova V.V., Sakhnov S.N. (2017). Ex vivo rabbit and human corneas as models for bacterial and fungal keratitis. Graefe’s Arch. Clin. Exp. Ophthalmol..

[31] Balagurunathan K., Nakato H., Desai U.R. (2014). Glycosaminoglycans: Chemistry and Biology.

[32] Wang X., Sun H., Mu T. (2024). Materials and structure of polysaccharide-based delivery carriers for oral insulin: A review. Carbohydr. Polym..

[33] Ahmadian E., Dizaj S.M., Eftekhari A., Dalir E., Vahedi P., Hasanzadeh A., Samiei M. (2020). The Potential Applications of Hyaluronic Acid Hydrogels in Biomedicine. Drug Res..

[34] Harmening N., lug M., Gramann K., Miklody D. (2022). HArtMuT-modeling eye and muscle contributors in neuroelectric imaging. J. Neural Eng..

[35] Hefft D.I., Adeutnji C.O. (2024). Chapter 8—Alginate in food and beverage formulations. Applications of Seaweeds in Food and Nutrition.

[36] Sun R.L., Jones D.B. (2007). Clinical characteristics and outcome of Candida keratitis. Am. J. Ophthalmol..

[37] Baldinger J., Doft B.H., Burns S.A., Johnson B. (1986). Retinal toxicity of amphotericin B in vitrectomised versus non-vitrectomised eyes. Br. J. Ophthalmol..

[38] Payne J.F., Keenum D.G., Sternberg P., Thliveris A., Kala A., Olsen T.W. (2010). Concentrated Intravitreal Amphotericin B in Fungal Endophthalmitis. Arch. Ophthalmol..

[39] Ongoren B., Kara A., Casettari L., Tiboni M., Lalatsa A., Sanz-Perez A., Gonzalez-Burgos E., Romero A., Juberias A., Torrado J.J. (2024). Leveraging 3D-printed microfluidic micromixers for the continuous manufacture of melatonin loaded SNEDDS with enhanced antioxidant activity and skin permeability. Int. J. Pharm..

[40] Bonneau N., otey A., Vitoux M.A., Magny R., Guerin C., Baudouin C., Peyrin J.M., Brignole-Baudouin F., Reaux-Le Goazigo A. (2023). Corneal neuroepithelial compartmentalized microfluidic chip model for evaluation of toxicity-induced dry eye. Ocul. Surf..

[41] Zhu Y., Zhu Y., Nasiri R., Davoodi E., Zhang S., Saha S., Linn M., Jiang L., Haghniaz R., Hartel M.C. (2023). A Microfluidic Contact Lens to Address Contact Lens-Induced Dry Eye. Small.

[42] Yamanaka T., Niino T., Omata S., Harada K., Mitsuishi M., Sugimoto K., Ueta T., Totsuka K., Shiraya T., Araki F. (2022). Bionic eye system mimicking microfluidic structure and intraocular pressure for glaucoma surgery training. PLoS ONE.

[43] Dai B., Zhang L., Zhao C., Bachman H., Becker R., Mai J., Jiao Z., Li W., Zheng L., Wan X. (2021). Biomimetic apposition compound eye fabricated using microfluidic-assisted 3D printing. Nat. Commun..

[44] Serrano D.R., Kara A., Yuste I., Luciano F.C., Ongoren B., Anaya B.J., Molina G., Diez L., Ramirez B.I., Ramirez I.O. (2023). 3D Printing Technologies in Personalized Medicine, Nanomedicines, and Biopharmaceuticals. Pharmaceutics.

